# Complete Resolution of a Large, Locally-advanced Cutaneous Squamous Cell Carcinoma with the Immune-modulating PD-1 Inhibitor Pembrolizumab

**DOI:** 10.7759/cureus.8072

**Published:** 2020-05-12

**Authors:** Cagney Cristancho, Ivy Riano, Daniel Guareras-Paredes, Robin Park, Kala Seetharaman

**Affiliations:** 1 Internal Medicine, Metrowest Medical Center/Tufts University School of Medicine, Framingham, USA; 2 Internal Medicine, Metrowest Medical Center/ Tufts University School of Medicine, Framingham, USA; 3 Hemato-Oncology, Metrowest Medical Center, Framingham, USA

**Keywords:** pembrolizumab, cscc, pd-1 inhibitors

## Abstract

Locally advanced cutaneous squamous cell carcinoma (cSCC) represents a challenge in treatment. Only very recently (February 2020) have guidelines been released regarding the management of unresectable, locally advanced cSCC. With the introduction of check point inhibitors during the last decade, anti-PD-1 antibodies represent a novel immunotherapeutic strategy in cancer. We present a case of an advanced cSCC not amenable to surgical resection, who experienced dramatic improvement following treatment with the programmed cell death protein 1 receptor (PD-1) inhibitor pembrolizumab as an immunotherapeutic strategy.

## Introduction

Cutaneous squamous cell carcinoma (cSCC) is the second most common cancer in the United States, behind only by basal cell carcinoma [1]. Numerous studies have shown a recent acceleration in the incidence of cSCC [2-3]. A number of risk factors are associated with development of cSCC, the most recognized of which is ultraviolet radiation. Chronic sun exposure, total site-specific exposure, and number of site-specific sunburns all strongly correlate with development of cSCC [4-5]. In line with this evidence are observations of higher cSCC rates in occupations involving outdoor work [5]. Actinic keratoses are sun-induced precancerous lesions, while Bowen’s disease refers to cSCC in situ. Both lesions, if left untreated, can progress to invasive cSCC with the potential for metastasis [6].

Local, uncomplicated disease is treated and often cured with surgical resection of the dysplastic tissue alone, using cutterage or electrodissection techniques. In cases of positive surgical margins containing dysplastic tissue, additional radiotherapy (RT) is often administered [7]. RT is also recommended for nonsurgical candidates and as adjuvant treatment for poorly vascularized or cartilaginous-area tumors, with extensive perineural involvement, but is not recommended for those individuals with genetic syndromes predisposing to increasing skin cancer risk (e.g. basal cell nevus syndrome), and relatively contraindicated for patients with connective tissue diseases (e.g. scleroderma) [7]. Systemic therapy is reserved for locally advanced (unresectable) or metastatic disease [8]. The choice of therapy remains a matter of debate and is often approached with multidisciplinary input.

The recent development of programmed cell death protein 1 receptor (PD-1) inhibitor immunotherapies has significantly advanced the treatment options available in oncology care. Not only is PD-1 inhibition effective, but PD-1 inhibitors tend to carry fewer overall side effects compared to conventional chemotherapy [9]. Nine PD-1 inhibitors are now approved by the FDA for the treatment of a variety of malignancies. The first of these was for advanced melanoma (2014), but now includes 16 other types of cancers [10]. Of most relevance, the PD-1 inhibitor cemiplimab was FDA-approved for cSCC in September 2018. Here, we present a dramatic example of successful treatment of a locally advanced, unresectable cSCC with the PD-1 inhibitor pembrolizumab.

## Case presentation

A 66-year-old man with no pertinent past medical history presented to oncology clinic with a 1-year history of a progressively enlarging rash on his left cheek. Physical examination revealed a large, ulcerative lesion located on his left face measuring approximately 12.5 x 13.5 cm. It extended superiorly to the level of the eyebrow and inferiorly to the level of his mouth. Medially it extended 1 cm from the lateral aspect of the nose. The lesion was erosive, with localized bleeding and purulent secretions. There were no signs of lymphadenopathy. A shave biopsy confirmed the diagnosis of a moderately-to-poorly differentiated invasive cSCC.

**Figure 1:**
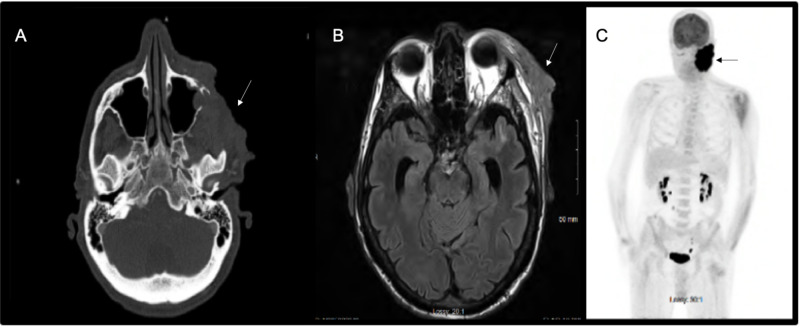
(A) Brain CT scan; (B) Brain MRI; (C) PET scan Radiographic workup of the lesion demonstrates (A) CT axial 8.9-cm mass with the invasion of the soft tissues of the left face, with involvement and bony destruction of the left zygomatic arch and the lateral wall of the left maxillary sinus; (B) MRI T2-FLAIR axial image demonstrating a mass in the left frontozygomatic region invading the lateral orbital region extraconal; (C) whole-body coronal PET scan demonstrating increased FDG-uptake in the left facial neoplasm CT, computed tomography; MRI, magnetic resonance imaging; PET, positron emission tomography

Computed tomography (CT) and MRI of the head and neck showed an 8.9-cm mass in the AP dimension (Figure 1A, 1B) with the invasion of the soft tissues of the left face, with involvement and bony destruction of the left zygomatic arch and the lateral wall of the left maxillary sinus. The mass extended into the left maxillary sinus and involved the extraconal soft tissues of the left orbit with possible involvement of the left lateral rectus muscle. There was a tumor in the infratemporal fossa and around the ramus of the mandible, with extensive enhancement after the administration of gadolinium contrast. There was no evidence of cervical lymphadenopathy.

Positron emission tomography (PET) scan showed intense FDG avidity associated with the mass. There was no evidence of metastatic disease (Figure 1C).

**Figure 2:**
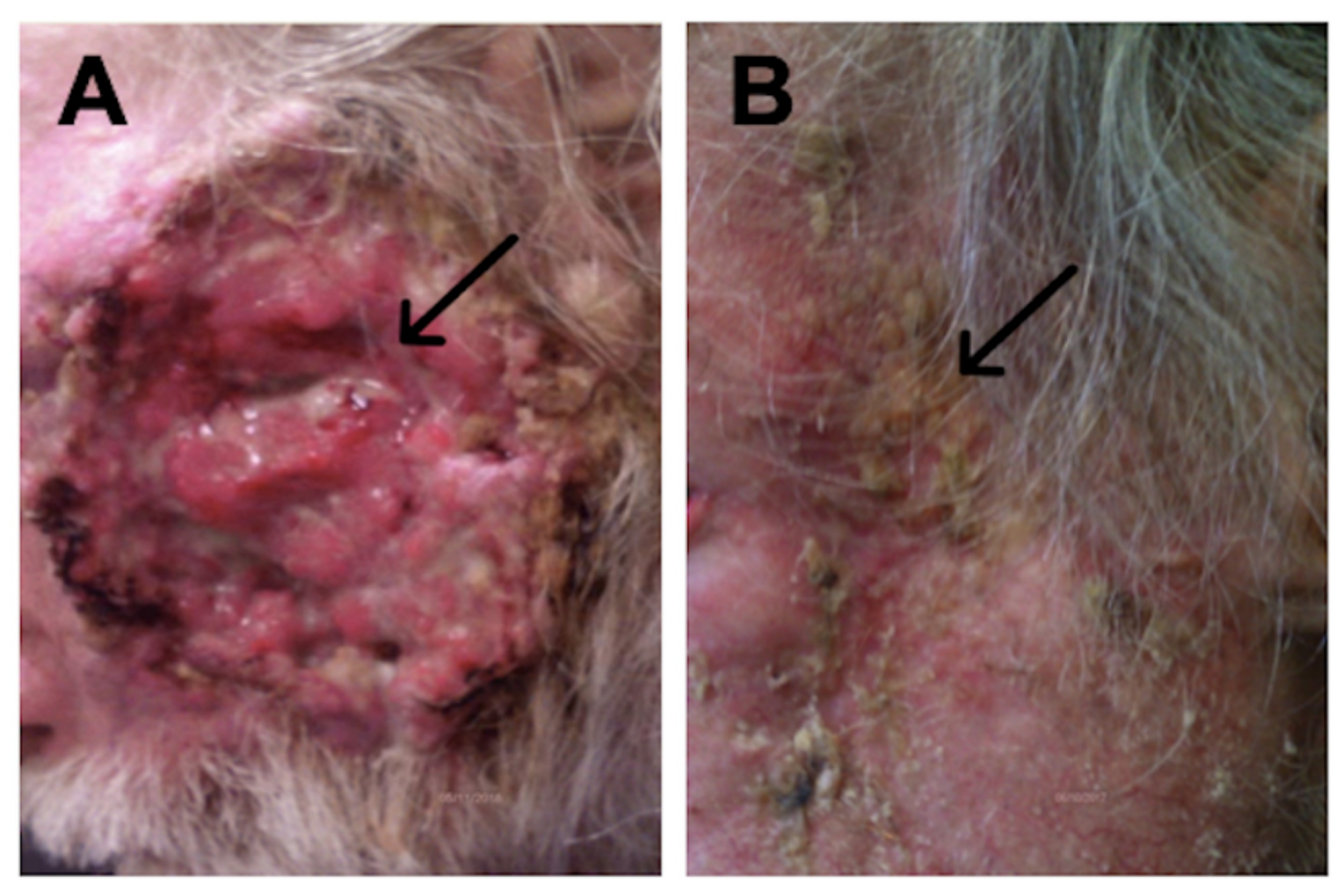
(A) Left: before therapy with pembrolizumab; (B) Right: after fifteen sessions of pembrolizumab

A regimen of pembrolizumab 200 mg IV every 3 weeks was initiated, with an initial plan for 2 years of treatment duration. The patient began to clinically response after the 4th session, with shrinkage of the tumor (Figure 2); no side effects were observed. The patient received a total of 15 sessions, with complete resolution of the tumor. There was no evidence of recurrence at one-year follow-up.

## Discussion

Locally advanced cSCC is a life-threatening condition that remains a significant therapeutic challenge. Prior to February 2020, few guidelines existed regarding an approach to management, which usually consisted of combinations of surgical excision where possible, systemic chemotherapy, and local radiation. Although these approaches continue to be first-line options in many circumstances, the FDA approval of the first PD-1 inhibitor for sCCC in September 2018 began to rapidly transform the field. In this case report we show how striking improvement can be achieved with PD-1 inhibition, and discuss an approach to management in cSCC in the context of recent European guidelines.

Prior to the introduction of PD-1 medications, the primary medical management of invasive cSCC often included a combination of epidermal growth factor (EGFR) receptor inhibitors, chemotherapeutics including platinum agents (usually cisplatin and carboplatin), and the fluoropyrimidine-based compound 5-FU. EGFR is highly expressed in cSCC and plays a crucial role in signal‐transduction pathways that regulate key cellular functions involved in cell proliferation, invasion, angiogenesis, and metastasis [8]. Available targeted EGFR inhibitors include antibody-based inhibitors of the extracellular domain of EGFR (cetuximab, panitumumab) and small-molecule tyrosine kinase inhibitors including erlotinib, gefitinib, and lapatinib [11]. Clinical trials have shown that anti-EGFR inhibitors have a moderate response often of short duration and their use is limited by adverse event profiles [12]. Most recent guidelines recommend anti-EGFR inhibitors as a second-line treatment as monotherapy or along with RT or chemotherapy for patients that are not candidates for PD-1 inhibitors.

Conventional chemotherapy regimens for cSCC consist of parenteral cytotoxic agents, usually bleomycin or cisplatin. Efficacy of electrochemotherapy in terms of disease control and local response has been reported in a range of 20%-70% of cases. Electrochemotherapy can also be used in cSCC to reduce tumor progression with the benefit of controlling bleeding and mass-related symptoms. A European multi-institutional prospective (EURECA) trial studied electrochemotherapy (bleomycin) for skin tumors, including 50 cSCC of the head and neck not suitable for surgery or chemotherapy/RT, as decided by multidisciplinary board. At 2-months follow-up, complete response was achieved in 55% of cSCC, partial response in 24%, stable disease in 15%, and progression in 4% [11]. However, despite chemotherapy being an option for patients who are not surgical candidates or those with metastatic disease, combination chemotherapies result in higher toxicity and lower patient compliance.

PD-1 is a surface protein expressed primarily on natural killer cells as well as those of the adaptive immune system (T- and B-cells). Its ligand, PD-L1/L2, is expressed on a multitude of tissues including tumor cells, where its successful binding to PD-1 prevents apoptosis and encourages immune exhaustion [13]. Therapeutic agents directed toward blocking this PD-binding interaction are efficacious both by encouraging tumor apoptosis as well as enhancing the effects of immune-mediated tumor cytotoxicity.

In 2017 in an expansion phase I study, which included 26 patients, cemiplimab at a dose of 3 mg/kg every 2 weeks intravenously a 50% response was observed. Results from a subsequent phase II study -which included 59 patients with metastatic disease- demonstrated an objective response of 47%, with an adverse events profile similar to other PD-1 inhibitors [14]. Based on these results, cemiplimab was FDA-approved in July 2018 and appears to offer both improved response rates while carrying fewer side effects when indirectly compared to conventional chemotherapies. Importantly, these trials excluded patients with organ transplants, hematologic malignancies, or medical conditions requiring immunosuppressives. Patients with these comorbidities are recommended to receive more conventional chemotherapies as described above [15].

At the time of patient’s presentation, no PD-1 inhibitors were FDA-approved for the treatment of cSCC, and no guidelines existed. The patient was evaluated by a multidisciplinary team consisting of dermatology, head and neck surgery, radiation oncology and medical oncology. His case was deemed non-operative due to the extensive local spread, and a course of systemic therapy was favored. The two options of treatment were chemotherapy with a combination of carboplatin or cisplatin with 5-fluorouracil and cetuximab, versus monotherapy with an immune checkpoint inhibitor. We ultimately favored a first-line PD-1 inhibitor trial based on the size and location of the lesion, as well as the elevated risk of infection with more conventional immunosuppressive chemotherapy. At the time, most published cases supported the use of PD-1 inhibitors only in cases of metastatic disease or as a second-line option with the failure of conventional chemotherapy. This case report highlights the occasional dramatic effect of PD-1 inhibition for the treatment of cSCC and it remains to be determined if this medication could outperforms cemiplimab in regard to efficacy or safety. Unpublished reports from a small (N=39) phase II clinical trial (CARSKIN, NCT02883556) study evaluating pembrolizumab as first-line monotherapy for unresectable cSCCs demonstrated robust antitumor activity regardless of PD-L1 expression levels and suggests similarly high efficacy compared to cemiplimab [16]. The lack of randomized, head-to-head comparisons of cemiplimab and pembrolizumab limit the ability to comment on the individualized risk:benefit anaylsis and approach to locally advanced or metastatic cSCC.

## Conclusions

Here we present a case of complete resolution of cutaneous SCC after 15 cycles of the PD-1 inhibitor pembrolizumab. This therapy was chosen based on the location of the tumor and comorbid high risk of infection. The recent European guidelines suggest first-line use of cemiplimab for non-operative cSCC. This unique case with no metastasis and no perineural involvement highlights a dramatic response to pembrolizumab monotherapy, resulting in complete resolution. This case adds to the literature regarding the efficacy of PD-1 inhibition with pembrolizumab for cSCC. 
